# CACHET-CADB: A Contextualized Ambulatory Electrocardiography Arrhythmia Dataset

**DOI:** 10.3389/fcvm.2022.893090

**Published:** 2022-07-01

**Authors:** Devender Kumar, Sadasivan Puthusserypady, Helena Dominguez, Kamal Sharma, Jakob E. Bardram

**Affiliations:** ^1^Department of Health Technology, Technical University of Denmark, Kongens Lyngby, Denmark; ^2^Department of Cardiology, Bispebjerg-Frederiksberg Hospital, Copenhagen, Denmark; ^3^U. N. Mehta Institute of Cardiology and Research Centre, Civil Hospital Campus, and SAL Hospital, Ahmedabad, India

**Keywords:** arrhythmias, context-aware ECG, wearable ECG, atrial fibrillation, ambulatory ECG, arrhythmia dataset

## Abstract

ECG is a non-invasive tool for arrhythmia detection. In recent years, wearable ECG-based ambulatory arrhythmia monitoring has gained increasing attention. However, arrhythmia detection algorithms trained on existing public arrhythmia databases show higher FPR when applied to such ambulatory ECG recordings. It is primarily because the existing public databases are relatively clean as they are recorded using clinical-grade ECG devices in controlled clinical environments. They may not represent the signal quality and artifacts present in ambulatory patient-operated ECG. To help build and evaluate arrhythmia detection algorithms that can work on wearable ECG from free-living conditions, we present the design and development of the CACHET-CADB, a multi-site contextualized ECG database from free-living conditions. The CACHET-CADB is subpart of the REAFEL study, which aims at reaching the frail elderly patient to optimize the diagnosis of atrial fibrillation. In contrast to the existing databases, along with the ECG, CACHET-CADB also provides the continuous recording of patients' contextual data such as activities, body positions, movement accelerations, symptoms, stress level, and sleep quality. These contextual data can aid in improving the machine/deep learning-based automated arrhythmia detection algorithms on patient-operated wearable ECG. Currently, CACHET-CADB has 259 days of contextualized ECG recordings from 24 patients and 1,602 manually annotated 10 s heart-rhythm samples. The length of the ECG records in the CACHET-CADB varies from 24 h to 3 weeks. The patient's ambulatory context information (activities, movement acceleration, body position, etc.) is extracted for every 10 s interval cumulatively. From the analysis, nearly 11% of the ECG data in the database is found to be noisy. A software toolkit for the use of the CACHET-CADB is also provided.

## 1. Introduction and Background

A heart arrhythmia like AF alone affects nearly 2% of the global adult population and is one of the major contributors to CVD related morbid conditions and mortality (1, 2). The management of AF includes anti-coagulation to prevent strokes and heart rhythm-modifier medications (3, 4). Also, therapies like electrophysiological pulmonary-vein isolation (PVI) can also be offered to selected and suitable candidates with good curative results (5). However, for treatment to be effective in preventing further complications, early diagnosis and timely evaluation of AF plays a vital role. Analysis of electrocardiogram (ECG) signals is a non-invasive and cost-effective way of diagnosing AF. Due to their transient nature, paroxysmal AF remains under diagnosed in baseline ECGs and require long-term ECG monitoring. However, long-term preemptive monitoring is challenging as manual analysis of days/weeks-long ECG needed for detecting paroxysmal AF is resource and time-consuming.

Over the years, many computer-based algorithms have been developed for faster and accurate detection of AF and other types of arrhythmias (6). More recently, with the advent of ML and DL, the field of computer-aided AF analysis has experienced a huge breakthrough (6–8). As compared to traditional ML and other feature engineering-based approaches, DL-based models can achieve end-to-end classification, thus removing the dependence on domain experts in the classification and stratification process. Despite all these advancements, one of the major challenge of using DL in AF classification is the availability of training and validation datasets. Although the DL algorithms can directly learn features from raw ECG data, it requires large and diverse datasets. The training data diversity helps the models to incorporate all the variations in inter/intra-personal ECG morphologies.

To meet this demand, many Internet ECG datasets such as the AFDB (9), MITDB (10), PTB-LX (11), CinCDB (12), OA-ADB (13), and DeepQ (14) have been published. Table 1 provides a summary of these publicly available arrhythmia databases. MITDB and AFDB are the earliest available ones and have been used extensively as a benchmark in training and evaluating ML/DL-based arrhythmia detection models (6, 7, 15).

**Table 1:** Technical specifications and ECG annotation statistics of publicly available ECG databases. Freq, sampling frequency (Hz); Ch, no. of ECG channels.

**Database**	**Ch**	**Freq** **(Hz)**	**No.** **samples**	**Sample** **length**	**Rhythm** **classes**	**No.** **subjects**	**Context**	**Remark**
AFDB (9)	2	250	23	10 h	4	25	✗	Continuous, controlled environment
MITDB (10)	2	360	48	30 min	15	47	✗	Continuous, controlled environment
NSRDB (17)	2	128	18	24 h	1	18	✗	Continuous, ambulatory
DeepQ (14)	1	250	897	5 min	8	299	✗	Intermittent, controlled environment
OA-ADB (13)	6	400	2,000	30 s	15	200	✗	Continuous, ambulatory, patient-operated
CinC2017 (12)	1	300	8,528	9–60 s	4	–	✗	Intermittent, patient-operated
CACHET-CADB	1	1,024	1602	10s	4	24	✓	Continuous, ambulatory, patient-operated

Although the aforementioned databases have made a significant contribution for developing and evaluating arrhythmia detection models; generalization and comprehensive performance evaluation of such models under free-living conditions remain questionable and face a number of significant challenges (6, 15, 16):

Firstly, as mobile and wearable technology is advancing, wearable ECG devices have become available for longitudinal arrhythmia screening under free-living conditions. However, the majority of the current databases are either collected in controlled in-hospital settings or, in some cases, under the environments where patients are sitting without any motion. Therefore, the recordings are relatively clean and lack the ECG morphology changes and confounding artifacts that occur under free-living conditions. When the classification models trained on these datasets are applied to ambulatory wearable-based ECG recordings, they result in non-trivial false positives due to the degradation in the signal quality (18).

Secondly, the patient's context, such as physical activity and posture change, food intake (drinks or heavy meal), or mental stress, are known to introduce morphological changes in the ECG signal (19, 20). Existing databases only provide the raw ECG data, while information on the patient's context during the recording is missing. Recent systematic literature reviews of computer-aided arrhythmia analysis highlight that the arrhythmia detection in an ambulatory setting remains challenging and prone to mis-classification, without understanding the patient's context in which the ECG was undertaken (6, 21). Even during a manual ECG analysis, whenever a cardiologist finds 10 or 30 s of ECG segment inconclusive, they often look for the longer context of the patient's ECG and rely on their knowledge about arrhythmia epidemiology (22). Therefore, the patient's ambulatory context is essential for avoiding inappropriate classification due to “arrhythmia mimicking artifacts.” Recent databases like DeepQ (14) have tried to address this problem by providing ECG recordings under the following three activity classes viz. sitting, walking, and lying down. These are, however, still a very limited set of activities and are recorded under circumstances that are very discordant from the real-world free-living ambulatory settings.

Thirdly, databases are usually generated from a single center for a short time period (minutes or hours) on a homogeneous group of participants. Due to large variations that exist in the morphologies of ECG waveforms and the lack of diversity in current datasets, models trained on such datasets result in a large number of false positives when applied to ECG from different user contexts, ethnic characteristics, anthropomorphic features, gender, age group, and time-periods (6, 23, 24). For instance, a multi-scale convolutional neural networks (23) showed a 98.18% accuracy when trained and validated on the AFDB, but its accuracy was reduced to 94.93% when applied on a Chinese dataset collected under free-living conditions. Similarly, the model by Andersen et al. (25) trained on AFDB has an excellent performance in 5-fold cross-validation on AFDB; however, it resulted in 4.9% FPR on previously unseen NSR database from healthy individuals.

To complement the existing databases and to address some of the above-mentioned challenges, we present the CACHET-CADB. In contrast to the existing databases, CACHET-CADB provides the following unique features:

- It contains longitudinal wearable based ECG data from arrhythmia patients collected under *free-living conditions*, thus suitable for training and evaluating algorithms aimed at enabling real-time ambulatory ECG monitoring of the patients.
- Along with the ECG dataset, it also provides *contextual data* such as activities, body positions, movement accelerations, patient-reported events like symptoms experienced, sleep quality, stress level, and food intake. This contextualized ECG data can help make the end-to-end DL-based ECG classification models more explainable. Further, identifying the algorithm's source of errors in relation to the patient's ambulatory context can help in dynamically fine-tune it for those false-positives prone/inducing contexts under free-living conditions.
- Is multi-site and diverse (currently, Denmark and India but will be expanded further).

Currently, the CACHET-CADB contains 259 days long contextualized ECG data from 24 patients. It also comprises 1,602 annotations of 10 s long ECG-waveform, manually annotated by two independent qualified cardiologists into four different heart rhythm classes: AF, NSR, “noise,” and “other.” The CACHET-CADB is under continuous development, and annotations by cardiologists will be added to the database as they become available. The ECG annotation tool will be made public to increase the effort of crowd-sourcing the annotation process. Along with the dataset, a set of Python scripts and other software tools for data access, visualization, and data processing are available on the CACHET GitHub repository (26). The dataset is freely available at DTU Data (27) at DTU.

## 2. Methods

This section explains the data acquisition process, including ethical considerations, the data collection methods and technology, the data specifications, and the annotation process.

### 2.1. Data Acquisition

#### 2.1.1. Ethical Consideration

The data for the CACHET-CADB was collected in India and Denmark. In Denmark, the study was exempted for ethical approval by the Danish Research Ethical Committee because the ECG recordings were only collected for technical purposes, and not to be used in a clinical setting (File # H-19071015). In India, the data collection was done with Mahatma Gandhi University of Medical Sciences and Technology (MGUMST), Jaipur, and the process complies with MGUMST's human participant's guideline and regulation as stated by the MGUMST Institutional Review Board (IRB). The approvals were granted on the ground that data collection was purely for technology development, and that the data would not be used for clinical diagnosis or treatment of the patients.

#### 2.1.2. Recruitment

The participants were recruited during their out-patient arrhythmia clinic visits *via* a general announcement to participate in the data collection study. It was also made clear to participants that their participation was purely for research purposes, and the collected data would not be used in their ongoing clinical diagnosis or treatment. Preference was given to the participants with a known history of paroxysmal AF or high AF risk factors. All participants signed an informed consent form and allowed their data to be used and shared publicly after subject identity anonymization.

#### 2.1.3. Data Collection Method

**Figure 1:**
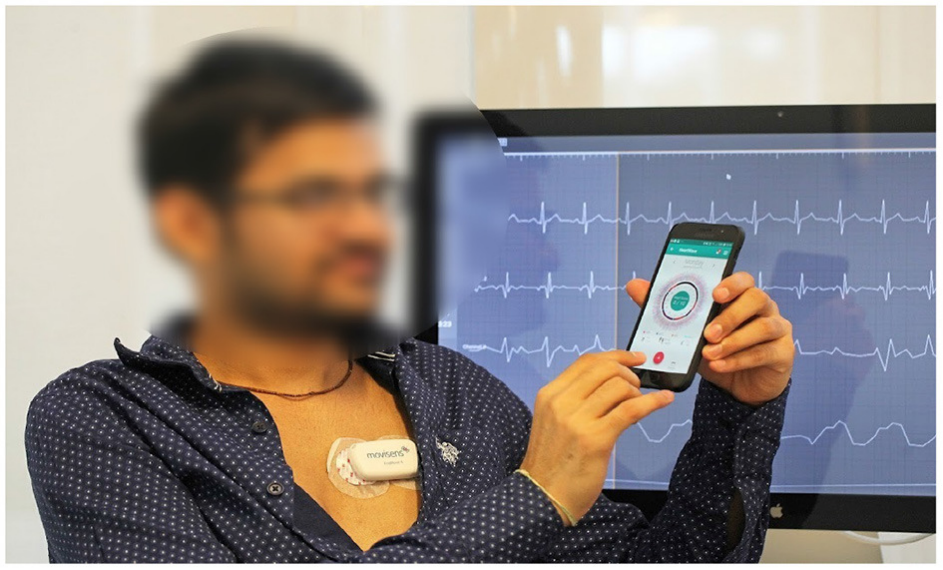
Data collection setup: (i) a chest-mounted single channel wireless ECG monitor collecting ECG and inertial (movement) measurements, and (ii) the mCardia mobile application for collection of patient-reported data (28).

We used the mCardia system (28) for the data collection. It uses a single-channel chest-mounted wireless ECG Holter [the Movisens ECGMove4 (29)] and a mobile application for data collection (Figure 1). Participants wore the ECG device using two disposable adhesive wet Ag/AgCl electrodes. All data was forwarded to, and stored in the CARP (30), which is a secure and scalable cloud-based infrastructure for health data science hosted at DTU. Each participant installed the mCardia mobile application on his/her phone and continuously wore the ECG device for a minimum of 24 h and up to 3 weeks. Participants were instructed to change the ECG electrodes daily and fill in the patient-reported information (symptoms, stress levels, sleep quality, and food intake) in the mCardia app. They were also instructed to take off the ECG device only for charging or during bathing/shower. Further details on the mCardia system and CARP can be found at https://carp.cachet.dk/mcardia/.

#### 2.1.4. Anonymization and Data Trimming

The initial recording length varied from 24 h to 3 weeks. For better manageability, analysis, and data handling, recordings were trimmed and assigned an anonymous ID (see **Figure 5**). In each record, the first (0th) and the last days are of variable lengths, whereas the rest are 24 h long, starting from midnight.

### 2.2. ECG Annotation Process

**Figure 2:**
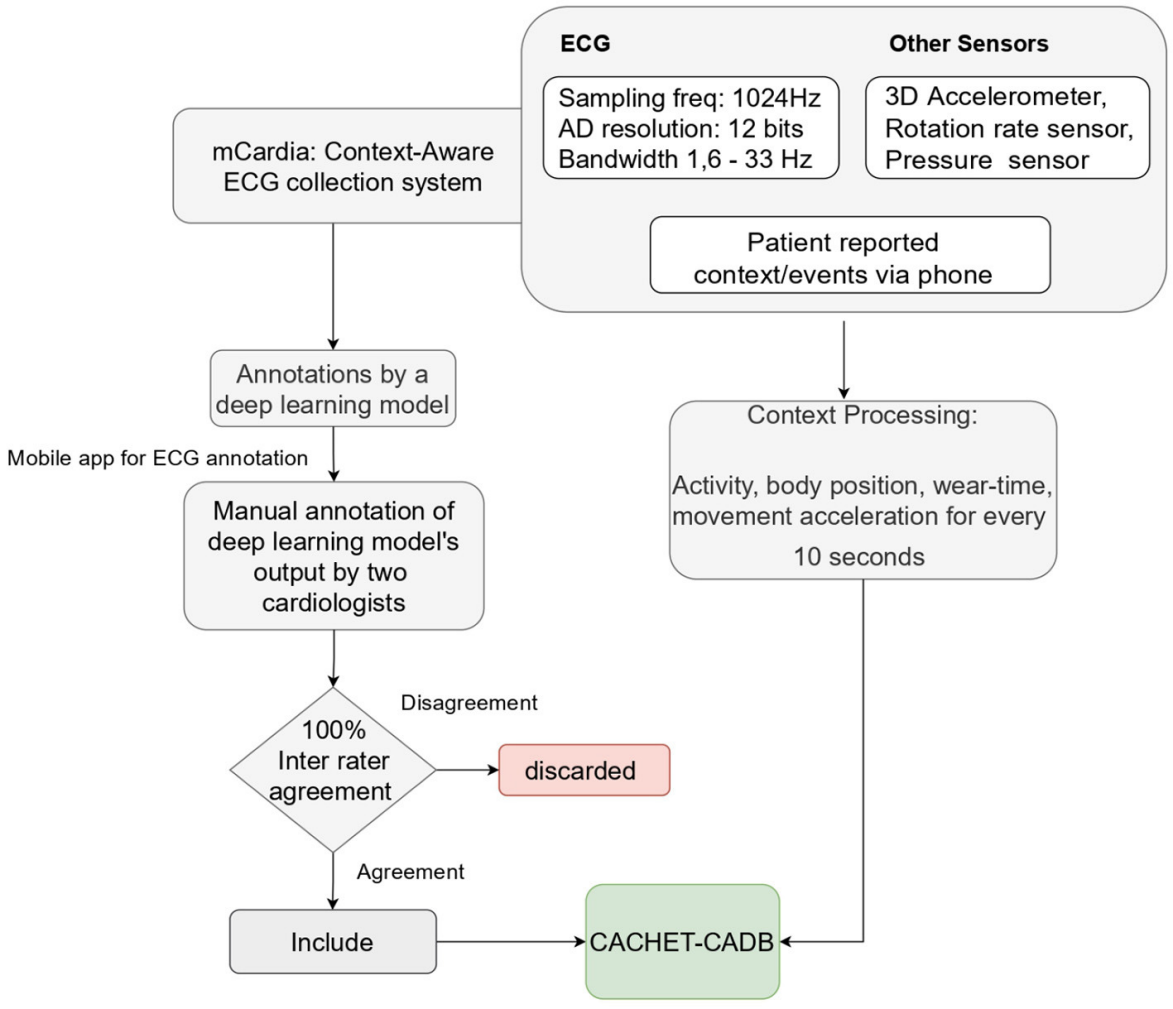
Overview of data collection and annotation process.

**Figure 3:**
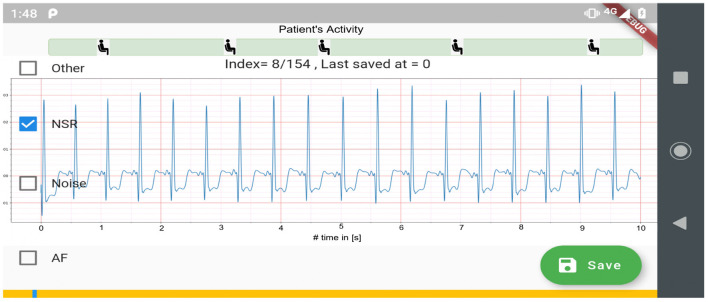
Mobile application used for ECG annotation.

Figure 2 shows the process used for annotating the ECG samples in the CACHET-CADB. A DL based AF detection model (25) was used to process the raw ECG recording. The AF onset and offset timestamps marked by the DL model were stored in CSV files. Thereafter, the segments between the onset and the offset were chopped into 10 s interval recordings and sent to two independent cardiologists for manual annotation *via* a mobile ECG annotation app. Figure 3 shows the user interface of the ECG annotation app used for the manual annotation. The annotation rules were discussed and agreed upon by the two cardiologists. A 10 s segment was assigned a label if it contained more than 50% of a particular rhythm type. If there were multiple rhythm classes in 10 s sample without having a majority (≥50%) of a particular class, then it was annotated as “others.” If artifacts in the 10 s signal precluded proper interpretation of the underlying rhythm, then the sample was annotated as “noise.” The annotations of the two independent cardiologists were compared for inter-observer agreement. If there were disagreement between the two cardiologists, the annotations were discarded. Thus, the final database only includes samples where there is an agreement between the two cardiologist's annotations.

### 2.3. Processing Contextual Data

The collected contextual data is of two categories: (1) patient-reported data collected *via* the mCardia app, and (2) sensor-generated data which is passively collected from the sensors on the mobile phone and the ECG Movisens device. Table 2 provides an overview of the types of collected data.

**Table 2:** Specifications of the collected data. S, sensed; PR, patient-reported; EB, event-based.

**Collected data type**	**Type**	**Data source**	**Sampling rate**
ECG	S	EcgMove4	1,024 Hz
3D acceleration	S	EcgMove4	64 Hz
Rotation rate sensor	S	EcgMove4	64 Hz
Pressure sensor	S	EcgMove4	8 Hz
Events	PR	EcgMove4 & Phone	EB
Sleep	PR & S	Phone	1/Day
Dietary	PR	Phone	1/Day

#### 2.3.1. Patient-Reported Data

Patient-reported contextual data was collected when the patient manually enters data during the study period. We collected two types of patient-reported context information; (1) experienced events, and (2) daily health reports. The events were registered by patients when they experienced any unusual symptoms (e.g., palpitations, heartburn, etc.) during the ECG recording period. It includes details about the type of symptom, its duration, activity during the symptom, and a short note providing more context and experience. Health reports were provided daily and comprised of a three short survey on meals (timings and type of meal (light, heavy, moderate), self-perceived stress level, and sleep quality (on a scale of 1–5). It should be noted that we only collected food intake timings and quantity (as light, heavy, or moderate) and not the specific details of what patients ate in their meals. The description of the meal itself was optional in the freestyle text input. The freestyle comments added by patients for further describing the symptoms or events were either in English or in the local vernacular language.

#### 2.3.2. Sensor-Generated Data

The sensed context is passively derived from the on-board sensors (3D acceleration sensor, gyroscope, and pressure sensor) of the chest-mounted Movisens ECG device and from the phone's sensors. Table 2 lists the sensors' sampling rates. The DataAnalyzer Tool (31) was used for processing data from the Movisens sensors, and context data such as movement acceleration, body position, activity, step count, wear time, energy expenditure, and MET levels were derived for an interval of 10 s. The movement acceleration, also known as MAI, is a typical physical activity metric that depicts bodily movements' intensity. The MAI is measured in “g,” which is multiples of Earth's gravity (1 g = 9.81 m/s^2^). In the DataAnalyzer Tool, the body positions were classified based on the inclination obtained from the 3D accelerometer. Its activity recognition is based on a white-box decision tree on the features extracted from the accelerometer and the barometric air pressure data (32). The type of recognized activities include unknown, lying, sitting, standing, cycling, slope up, jogging, slope down, walking, and not-worn. Similarly, the body positions are classified based on the inclination obtained from the 3D accelerometer. The body position classes include unknown, lying supine, lying left, lying prone, lying right, upright, sitting/lying, standing, and not-worn.

## 3. Data Records

**Figure 4:**
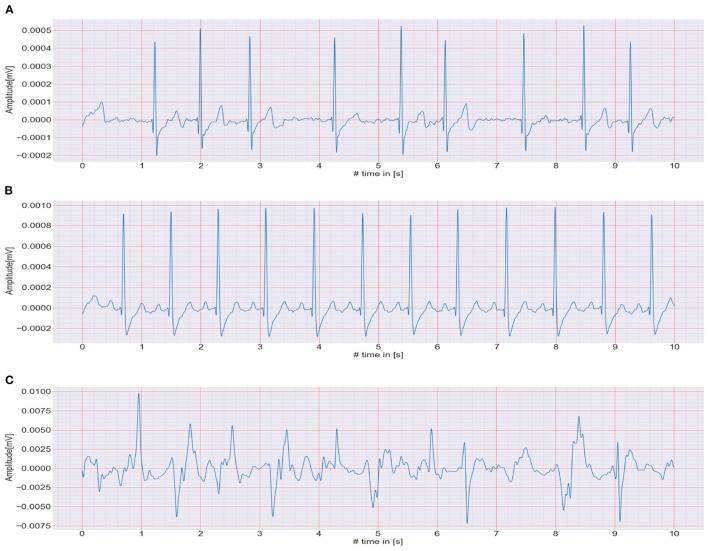
**(A–C)** Show the 10 s ECG recordings of AF, NSR, and Noise classes, respectively.

The CACHET-CADB includes over 259 days of single-channel contextualized ECG recording from 24 patients previously diagnosed with or suspected of the high risk of AF. Besides the patient's ambulatory contexts, it also contains 1,602, 10 s long annotation samples of 4 different ECG rhythm classes, namely, AF, NSR, noise, and others (anything excluding AF, NSR, and noise). A sample of each of these rhythm classes is shown in Figure 4. The CACHET-CADB is freely available on DTU Data figshare (27) under the name “CACHET-CADB.”

**Figure 5:**
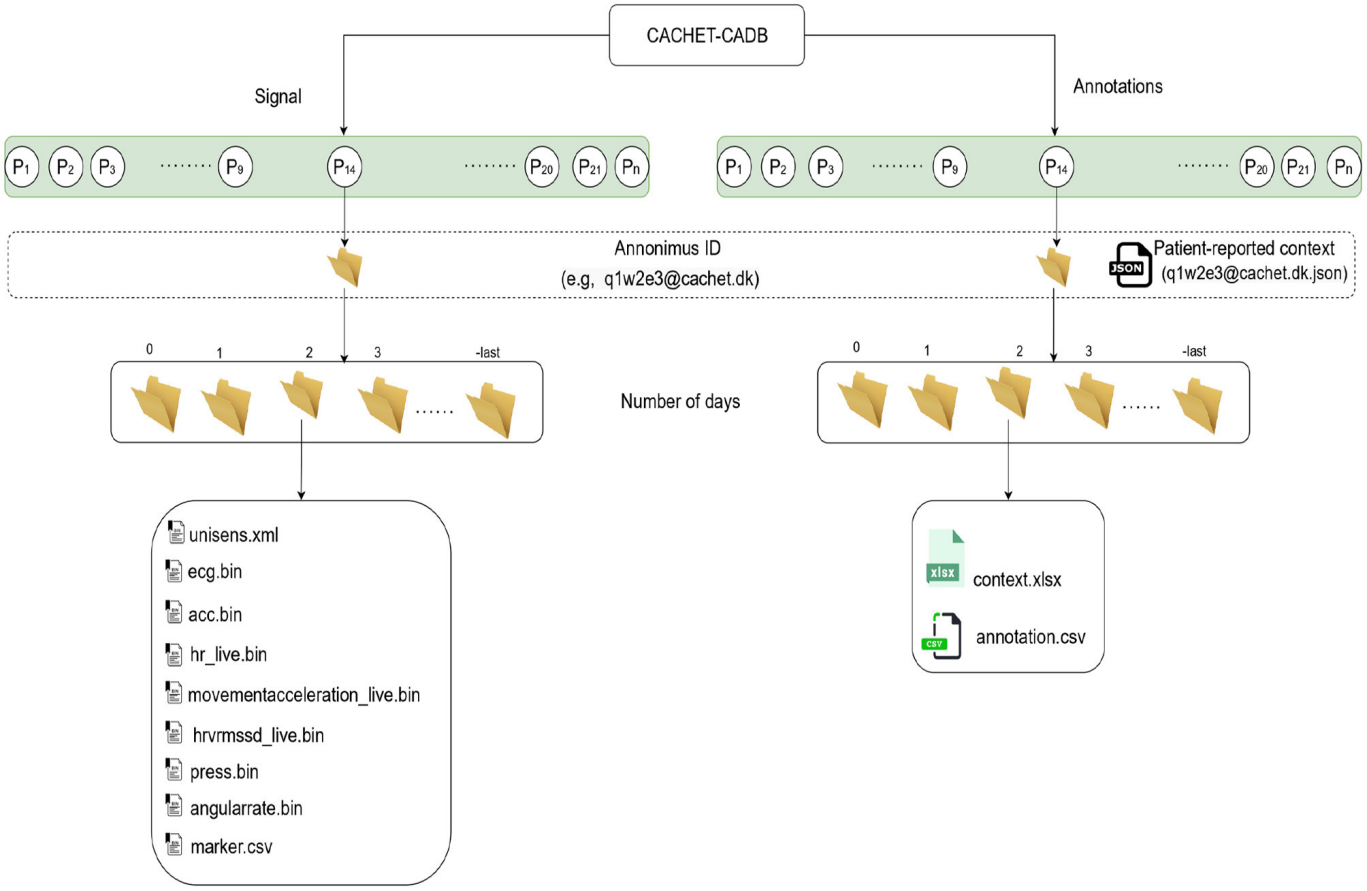
The structure of the data records in CACHET-CADB. Overall, the database is divided into two major parts; (i) the raw recordings in binary files and (ii) the contextual information including patient-reported data and the annotations. Each record is organized according to patient ID first and day in study subsequently.

Figure 5 describes the organization of the records in CACHET-CADB. For better manageability and incorporation of future updates, the dataset is split into two main parts: (i) the raw signals (i.e., ECG, 3D accelerometer, angular rate) and (ii) the annotations, while keeping the same folder structure inside each part. At the time of drafting this manuscript, the dataset has 24 records, spanning 259 days of recording from 24 patients of which, 7 were Danish and 17 were Indian. There were 15 males/9 females—with an average age of 59, and of which 11 patients had documented one or more AF episodes in past.

### 3.1. Raw Signals and Metadata

The raw sensor data is stored in Unisens (33) file format. It allows simultaneously multi-sensor data, with synchronous storage at different sample rates, and comes with a human-readable meta-file in XML format. As illustrated in Figure 5, for each day the *unisens.xml* file contains the metadata for the raw signals. Table 3 describes these metadata in detail. The general metadata information includes the start timestamp, the total recording time (in seconds), and the anonymous user id (same as the anonymous id for the entire recording). The patient metadata includes height, weight, gender, location of the ECG sensor, and age at the time of recording. The raw ECG, 3D accelerometer, angular rate, and pressure signals are in the *ecg.bin, acc.bin, angularrate.bin*, and *press.bin* files, respectively. To allow for any future processing and analysis of the recordings, the dataset contains the raw signal without any preprocessing or filtering.

**Table 3:** Metadata for the signal files described in the *unisens.xml* file of each record.

**Type**	**Key**	**Data type**	**Channel name**	**Description**
	Duration	String		Total recording time in seconds
General	Timestamp Start	String		Recording start time
	Measurement Id	String		Anonymous user id
	Height	String		Height in centimeters
	Weight	String		Weight in kilograms
Patient and	SensorVersion	String		Recording device version
Device	SensorType	String		Recording device type
	Age	String		Age at recording in years
	SensorLocation	String		ECG sensors location on body
	PersonId	String		Anonymous user id
	Gender	String		Gender (M/F)
ECG	ECG.bin	Binary	ECG I	Resolution: 12 bit, Input range CM = 560 mV, DM = ±5 mV, 3 db Bandwidth 1.6–33 Hz Output rate: 1,024 Hz
Accelerometer	Acc.bin	Binary	accX, accY, accZ	3D acceleration sensor Measurement range: ±16 g Output rate: 64 Hz
Angular Rate	Angularrate.bin	Binary	AngularRateX, AngularRateY, AngularRateZ	Rotation rate sensor: Measurement range: ±2,000 dps Output rate: 64 Hz
Pressure	Press.bin	Binary	Press	Measurement range: 300–1,100 hPa Noise: 0.03 hPa Output rate: 8 Hz
Marker	Marker.csv	Integer		Contains indexes of *events* when the patient experienced unusual systems and tapped on ECG Holter. Divide the index by 64 to get the event time in seconds from the start of the recording. Output rate: 64 Hz

However, given the recordings' ambulatory nature, any use of the data would probably need to implement baseline correction and removal of other artifacts beyond the normal ECG band [0.5–50 Hz].

### 3.2. Annotations and Metadata

As shown in Figure 5, the annotations follow the same folder structures as the raw signals. For each day, the *context.xlsx* and *annotation.csv* files contain the contextual and annotation data, respectively.

The *context.xlsx* file contains the patient's ambulatory context for every 10 s interval. These contextual data are derived from a 3D acceleration sensor, gyroscope, and pressure sensor, as described earlier. Table 4 provides the metadata for these contextual data, where the attributes listed in the table are columns in the *context.xlsx* file. The “unit” column in Table 4 represents the measurement unit of each attribute. The remark column provides the label of each subclass within the same column. For instance, *ActicityClass* has several sub-classes, such as lying, sitting/standing, cycling, slope up, or jogging. The corresponding subclass code (0, 1, 2...) represents them in the activity column of the *context.xlsx* file. Patient-reported data is provided as a single JSON file in each annotation folder (see Figure 5). The JSON file contains two types of data “*dailyInfo”*, and “*event”*. Their metadata are described in Tables 5, 6, respectively.

**Table 4:** Contextual-data descriptor table.

**Attribute**	**Unit**	**Remark**
Time rel	[s]	Relative time from start of measurements in seconds
Day rel	[d]	Number of days from start of measurement
Time rel	[hh:mm:ss]	Relative time from start of measurement
Date abs	[yyyy-mm-dd]	Absolute date
Time abs	[hh:mm:ss]	Absolute time
ActivityClass	–	Activity Class (0 = unknown, 1 = lying, 2 = sitting/standing, 3 = cycling, 4 = slope up, 5 = jogging, 6 = slope down, 7 = walking, 8 = sitting/lying, 9 = standing, 10 = sitting/lying/standing, 11 = sitting, 99 = not worn)
ActivityEnergyExpenditure	[kcal/d]	Activity energy expenditure (AEE) in kcal/d
Altitude	[m]	Altitude from barometer
BodyPosition	–	Body position (0 = unknown, 1 = lying supine, 2 = lying left, 3 = lying prone, 4 = lying right, 5 = upright, 6 = sitting/lying, 7 = standing, 99 = not worn)
InclinationDown	[deg]	Inclination of sensor axis down against the vertical (0–180°)
InclinationForward	[deg]	Inclination of sensor axis forward against the vertical (0–180°)
InclinationRight	[deg]	Inclination of sensor axis right against the vertical (0–180°)
MET		MET value directly calculated from regression models
MovementAcceleration	[g]	MovementAcceleration: Raw acceleration, bandpass filtered, vector magnitude
NonWearSleepWake	–	Sleep/Wake detection (0 = wake, 1 = sleep, 2 = not worn)
NonWearTime	–	Non wear detection (0 = worn, 1 = not worn)
StepCount	[steps]	Count of steps per output interval
TotalEnergyExpenditure	[kcal/d]	Total energy expenditure (TEE = BMR + AEE)
VerticalSpeed	[m/s]	Vertical speed, calculated from barometer

*The attributes are the columns of the context.xlsx file in the annotation folder of each day*.

**Table 5:** Metadata of patient-entered context data “*dailyInfo”* in JSON file.

**Field name**	**Description**
Date_time	Day for which the “*dailyInfo”* is filled
Bed_time	Bed time
Awake_time	Wake up time
Sleep_quality	Self-assessed sleep quality (1–5)
Stress_level	Self-assessed stress level (1–5)
Lunch_time	Lunch time
Lunch_weight	Lunch quantity (heavy, moderate, light)
Breakfast_time	Breakfast time
Breakfast_weight	Breakfast quantity (heavy, moderate, light)
Dinner_time	dinner time
Dinner_weight	Dinner quantity (heavy, moderate, light)
Other_time	Time of any other meal/drink
Other_weight	Meal/Drink quantity (heavy, moderate, light)

**Table 6:** Metadata of patient-entered “*event”* field in JSON file representing patient-reported symptoms that the patient may have experienced during the recording period.

**Field name**	**Description**
Id	Unique id
Notes	Note describing the unusual experience/symptoms
Labels	n/a
Source	How was the event entered? *"Tap"*: By tapping on the ECG Holter *"Self input"*: Manually created in the app
Deleted^1^	Was the event Deleted? (true/false)
Comments	n/a^2^
Duration	Time in seconds for which symptoms lasted
Symptom	Symptom experienced during the unusual event (e.g., “Dizziness”)
Activity	Patients activity when the unusual symptoms were experienced
Completed	Were the details of an event filled in? True: All fields were completed. False: Not filled/ Partially filled
Reviewed	n/a
Date_time	Time of the event as experienced by the patient

1 The patient could delete an event, e.g., if it was created by accidentally tapping the ECG device.

2 The patient's comments are removed for anonymity.

The *annotaion.csv* file contains the cardiologists' annotation of hearth rhythms. It contains the following columns: (i) the start index of 10 s long segment (*Start*), (ii) the end index of 10 s long segment (*End*), and (iii) the ECG rhythm class (*Class*). Table 7 provides the statistics of each of the annotated rhythm classes and their associated code in the *Class* column of the *annotation.csv* file.

**Table 7:** ECG annotation overview showing the class of rhythm types, its code in the *annotation.csv* file, and the number of available annotations for each class.

**Class**	**Code**	**#**
AF	1	747
NSR	2	615
Noise	3	221
Others	4	19

## 4. Technical Validation

### 4.1. Quality Assessment of ECG Annotation

Although the DL models (25) was used for automatic labeling (Figure 3), to ensure the quality and integrity of the rhythm annotation, we have released only the annotations that have been manually checked by the two independent cardiologists. A 100% inter-rater agreement policy is followed. The ECG segments on which there was a disagreement between two cardiologists are not included in this release.

### 4.2. Signal Quality Assessment

**Figure 6:**
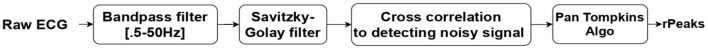
Analysis of ECG quality, QRS complex, and R-peak detection.

For testing the validity of the collected ECG data, an ECG signal quality assessment was done using an auto-correlation-based noise detector. Subsequently, the Pan Tomkinson algorithm (34) was used to calculate QRS complex/R-peaks. The steps used in the validation process are shown in Figure 6. As the ambulatory ECG signal tends to get contaminated by noise and other artifacts, first, a band-pass [0.5–50 Hz] filter was applied, and the baseline was removed. A Savitzky-Golay (35) filter followed this to smoothen out the data. Thereafter, the signal was chopped into 10 s long windows, and an auto-correlation based noise detector was applied to detect the noisy signal. Finally, the Pan Tomkinson algorithm (34) was used to calculate the QRS complexes and the R-peaks for each of these 10 s windows. Table 8 shows the number of R-peaks detected and the percentage of the noisy signal detected in each record. It should be noted that the discrepancy in the ECG noise percentage between patients (or within the same patient for different days) depended on factors such as how diligent the patients (or, in some cases, their caretakers) were in timely changing the adhesive ECG electrodes. In the ECG signal, intervals between the R-peak indicate heart rhythm's regularity. These RR intervals (RRI) features have been extensively used in DL-based AF detection models (25).

**Table 8:** Signal quality assessments and detection of QRS complex/R-peaks. Non-wear Time: Time for which device was taken off (for changing, bathing, for any other reasons).

**Record**	**User Id**	**Days**	**No. of R-peaks**	**Signal duration** **(hours)**	**Noisy signal** **(%)**	**Non-wear time** **(hours)**
P1	a2b3c4@cachet.dk	12	1,158,069	241.58	7.48	6.15
P2	t1y2u3@cachet.dk	7	673,950	139.40	6.47	1.03
P3	q1w2e3@cachet.dk	15	1,440,323	315.77	8.40	41.08
P4	p1q2w3@cachet.dk	8	739,199	173.14	5.80	10.85
P5	b1t2s3@cachet.dk	8	665,666	147.97	16.27	25.50
P7	k9v3r7@cachet.dk	12	913,892	260.16	12.43	41.91
P6	s1a2n3@cachet.dk	12	1,241,040	257.34	3.26	8.82
P8	g4v3r7@cachet.dk	22	2,895,927	479.16	9.90	77.98
P9	c1x2p3@cachet.dk	12	921,713	247.78	29.61	82.21
P10	k1x2p3@cachet.dk	16	1,297,163	359.85	31.72	80.28
P11	v2c3r4@cachet.dk	16	1,363,671	326.96	12.72	61.26
P12	r4p2n8@cachet.dk	14	1,988,086	308.91	6.88	6.31
P13	f7c4n6@cachet.dk	19	1,964,554	412.19	2.65	16.63
P14	j4y9x6@cachet.dk	12	1,035,832	262.94	29.90	111.36
P15	u3h6c1@cachet.dk	14	1,385,906	315.49	28.05	79.08
P16	i6t2v4@cachet.dk	17	1,567,938	359.86	6.29	25.71
P17	z2y4b9@cachet.dk	15	1,280,062	325.34	6.02	19.18
P18	g2v5x7@cachet.dk	5	431,256	92.95	3.23	1.54
P19	m1t2a3@cachet.dk	4	272,549	75.22	3.59	2.51
P21	y1t2r3@cachet.dk	8	778,148	168.93	10.34	12.10
P23	m1n2b3@cachet.dk	7	762,802	160.54	7.24	6.33
PNSR-1	deku_test@cachet.dk	1	105,079	24.00	0.49	0.56
PNSR-3	j5f3c2@cachet.dk	1	92,134	26.44	27.14	0.00
PNSR-4	w1y3n2@cachet.dk	2	191,867	48.00	5.63	2.05
Total		259	25,166,826	5529.94		726.57

Although we did identify noise in the dataset, we did not exclude the noise from the database. This was done intentionally to allow the CACHET-CADB to reflect a realistic distribution of ECG quality as expected under free-living conditions. ECG riddled with confounding artifacts and varying signal quality is expected when performing longitudinal ambulatory arrhythmia screening. Therefore, we put forward the CACHET-DB as a resource for designing and evaluating DL-based arrhythmia detection algorithms, which work under free-living condition without generating false positives. Moreover, the database can be used for creating unsupervised learning methods, which can enable feature extraction representing ECG quality variation in ambulatory settings. As already discussed, one of the main challenges with the existing arrhythmia ECG datasets is that they are collected in a clinically controlled environment and are relatively clean. Models trained on such clean datasets may result in many false-positive cases when applied on ECG collected under free-living conditions that inevitably has low signal quality and many artifacts (36, 37).

## 5. Discussion

This paper presents the design and development of a contextualized ECG database to support the development and generalization of ECG analysis and arrhythmia detection models. The CACHET-CADB has been developed as a part of the REAFEL (38) research project, which focuses on building mHealth and DL-based solutions for optimizing diagnosis of AF in the frail and elderly population. CACHET-CADB is particularly important for researchers who are working on bringing ECG analysis and AF detection on patient-operated wearable ECG into widespread adoption under free-living conditions. The database will be further expanded with more recordings and ECG annotation as they become available by following the data annotation and storage setup described above.

The ability to bring arrhythmia detection models in widespread adoption under free-living conditions is limited by the lack of a patient-operated ambulatory ECG dataset that truly represents all the confounding contamination expected in such conditions. The models trained on benchmark datasets in Table 1 show high performance when tested on the same datasets or similar datasets collected under clinical supervision. However, the high classification performances often obtained on these datasets are not reproducible when applied to patient-operated ECG data under free-living conditions. The patients-operated wearable-based ECG under free-living condition is often contaminated with arrhythmia mimicking artifacts and suffers from poor signal quality. The cause of the poor performance under free-living conditions is attributed to the lack of diversity and relatively good signal quality of ECG wave forms in these benchmark databases (18).

With wearable technology advancements, single lead portable ECG monitoring has been gained attraction for arrhythmia screening under free-living conditions (39). Coupling portable patient-operated ECG monitoring with computer-aided ML and DL-based classification algorithms can help in real-time and cost-effective longitudinal arrhythmia screening under free-living conditions. To achieve high sensitivity and reproducibility under free-living conditions, the CACHET-CADB provides an opportunity to train and evaluate arrhythmia detection models on a dataset representing all the ECG morphology changes and confounding noise contamination expected in free-living conditions.

### 5.1. Context-Aware ECG for Explainable DL Models

One advantage of CACHET-CADB over the existing database is the availability of patients' ambulatory context corresponding to the recorded ECG. In the absence of patients' context, the ECG analysis under free-living conditions is prone to mis-classification and misinterpretation (6). The contextual data can also be used for multi-model input and context-based heuristics to dynamically fine-tune the models' sensitivity and specificity under different user contexts in ambulatory settings. To reduce the FPR, algorithms should be made adaptive to the user's context—i.e., the sensitivity and specificity of algorithms should be dynamically adjustable. For instance, in the elderly population, there is a significantly higher prevalence of falls in patients with AF (40). Suppose an algorithm is applied to elderly patients' data and if a fall is detected, then the algorithm should factor-in for the fall in the dynamic adjustment of its sensitivity and specificity. Similarly, information about AF triggering contexts (41) such as high stress-level, food-intake (heavy meal), drinks (alcohol, caffeine) can be utilized to make algorithms more sensitive in those contexts.

**Figure 7:**
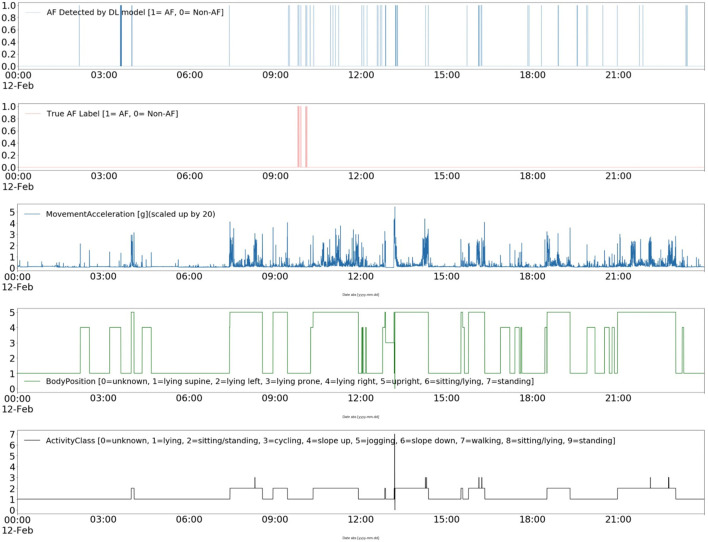
Explainable deep learning: This Figure shows a contextualized view of a deep learning-based AF detection model Andersen's (25) performance on a single day of ECG from CACHET-CADB. In 24 h of ECG under free-living conditions, short segments of false positive in a model's output are linked to change in activity, change in body position, and sudden movement accelerations.

Furthermore, the contextual data can pave the way for improving the interpretability of ML and DL models (42). For instance, Figure 7 shows a DL model's AF classification results, the “ground truth” annotations, and patient's ambulatory contexts (body position, activities, movement acceleration) for 24 h long record in CACHET-CADB. It can be inferred from Figure 7 that the model is resulting in more FP whenever there is a change in activity, body position, and movement acceleration, which is most prominent after 09:00 o'clock. Such information can be made available to a cardiologist for the manual inspection of the dataset thereby providing a better insight into when and why the AF detection algorithm has identified an AF episode. The information can also be utilized to build post-processing heuristics around these FP prone ambulatory contexts (43). With CACHET-CADB, we aim to provide the DL research community rich longitudinal contextualized ECG data that can help build and evaluate models which realistically work on patient-operated ECG from free-living ambulatory conditions.

### 5.2. Wearable ECG in Arrhythmia Monitoring and Its Economic Implications

The CACHET-CADB database is collected using the mCardia (28) system in the REAFEL (38) project, and its cost is comparable to other wearable-based single channel ECG devices. The main focus of the REAFEL study is to diagnose atrial fibrillation from patient-operated wearable ECG, away from highly controlled clinical environments, and thereby to make an accessible diagnostic tool for vulnerable populations who have difficulties in accessing to such clinically controlled measurements. Already, ambulatory wearable ECG has been found to be cost-effective in the detection of AF and reducing unnecessary hospital visits (44). However, there are significant potential economic gains in reducing manual examination of longitudinal ambulatory ECG by using automated arrhythmia detection algorithms. As pointed out by Wu et al. (14), the lack of sizable annotated and diverse ECG wearable datasets for testing and evaluating is one of the leading causes behind non/slow improvements in classification algorithms' performance. By making available the CACHET-CADB, we aim to help researchers to develop and evaluate algorithms for patient-operated wearable ECG, thereby making longitudinal ambulatory monitoring more economically robust and feasible.

## 6. Usage Notes

The design, data-descriptor, and the software tools for using CACHET-CADB are presented and made available for public use. When using this database, please cite the current publication. The new data recording and ECG annotations on the existing records will be added to CACHET-CADB periodically when they become available; details of the subsequent release will be available at CACHET's website (45).

## 7. Code Availability

Visual inspection and editing of records can be done using the UnisensViewer tool http://software.unisens.org/download/UnisensViewer/UnisensViewer_Setup.exe. Python library *pyunisens* (https://github.com/Unisens/pyunisens) can be used for reading and editing the signal programmatically. We also provide a basic code example and Jupyter Notebook in Python for using the database https://github.com/cph-cachet/cachet-ecg-db. The contextual data file context.xlsx can be loaded and viewed using the panda library (https://pandas.pydata.org/); an example code for the same can be found at https://github.com/cph-cachet/cachet-ecg-db. All software is open-sourced under an MIT license, and we welcome pull requests.

## Data Availability Statement

The datasets presented in this article can be found in online repositories. The names of the repository/repositories and accession number(s) can be found at: https://doi.org/10.11583/DTU.14547264.

## Ethics Statement

The studies involving human participants were reviewed and approved by Danish Research Ethical Committee and Mahatma Gandhi University of Medical Sciences and Technology (MGUMST), Jaipur India. The patients/participants provided their written informed consent to participate in this study.

## Author Contributions

DK and JB conceived the database and implemented the technology for data collection and storage. DK conducted the data collection and manual ECG annotation process in collaboration with HD and KS. DK analyzed the data and wrote the Python scripts. DK, JB, and SP wrote the paper. JB obtained the funding. All authors reviewed the manuscript and contributed to the article and approved the submitted version.

## Funding

This research has been funded by the Innovation Fund Denmark as part of the REAFEL project (IFD Project No. #6153–00009B) and the Copenhagen Center for Health Technology.

## Conflict of Interest

The authors declare that the research was conducted in the absence of any commercial or financial relationships that could be construed as a potential conflict ofinterest.

## Publisher's Note

All claims expressed in this article are solely those of the authors and do not necessarily represent those of their affiliated organizations, or those of the publisher, the editors and the reviewers. Any product that may be evaluated in this article, or claim that may be made by its manufacturer, is not guaranteed or endorsed by the publisher.
